# In the Pursuit of Privacy: The Promises and Predicaments of Federated Learning in Healthcare

**DOI:** 10.3389/frai.2021.746497

**Published:** 2021-10-06

**Authors:** Mustafa Y. Topaloglu, Elisabeth M. Morrell, Suraj Rajendran, Umit Topaloglu

**Affiliations:** ^1^ Wake Forest University, Winston Salem, NC, United States; ^2^ Wake Forest School of Medicine, Winston Salem, NC, United States

**Keywords:** machine learning, federated learning, data sharing, machine learning legal and ethical issues, machine learning social and regulatory issues

## Abstract

Artificial Intelligence and its subdomain, Machine Learning (ML), have shown the potential to make an unprecedented impact in healthcare. Federated Learning (FL) has been introduced to alleviate some of the limitations of ML, particularly the capability to train on larger datasets for improved performance, which is usually cumbersome for an inter-institutional collaboration due to existing patient protection laws and regulations. Moreover, FL may also play a crucial role in circumventing ML’s exigent bias problem by accessing underrepresented groups’ data spanning geographically distributed locations. In this paper, we have discussed three FL challenges, namely: privacy of the model exchange, ethical perspectives, and legal considerations. Lastly, we have proposed a model that could aide in assessing data contributions of a FL implementation. In light of the expediency and adaptability of using the Sørensen–Dice Coefficient over the more limited (e.g., horizontal FL) and computationally expensive Shapley Values, we sought to demonstrate a new paradigm that we hope, will become invaluable for sharing any profit and responsibilities that may accompany a FL endeavor.

## Introduction

**FIGURE 1:**
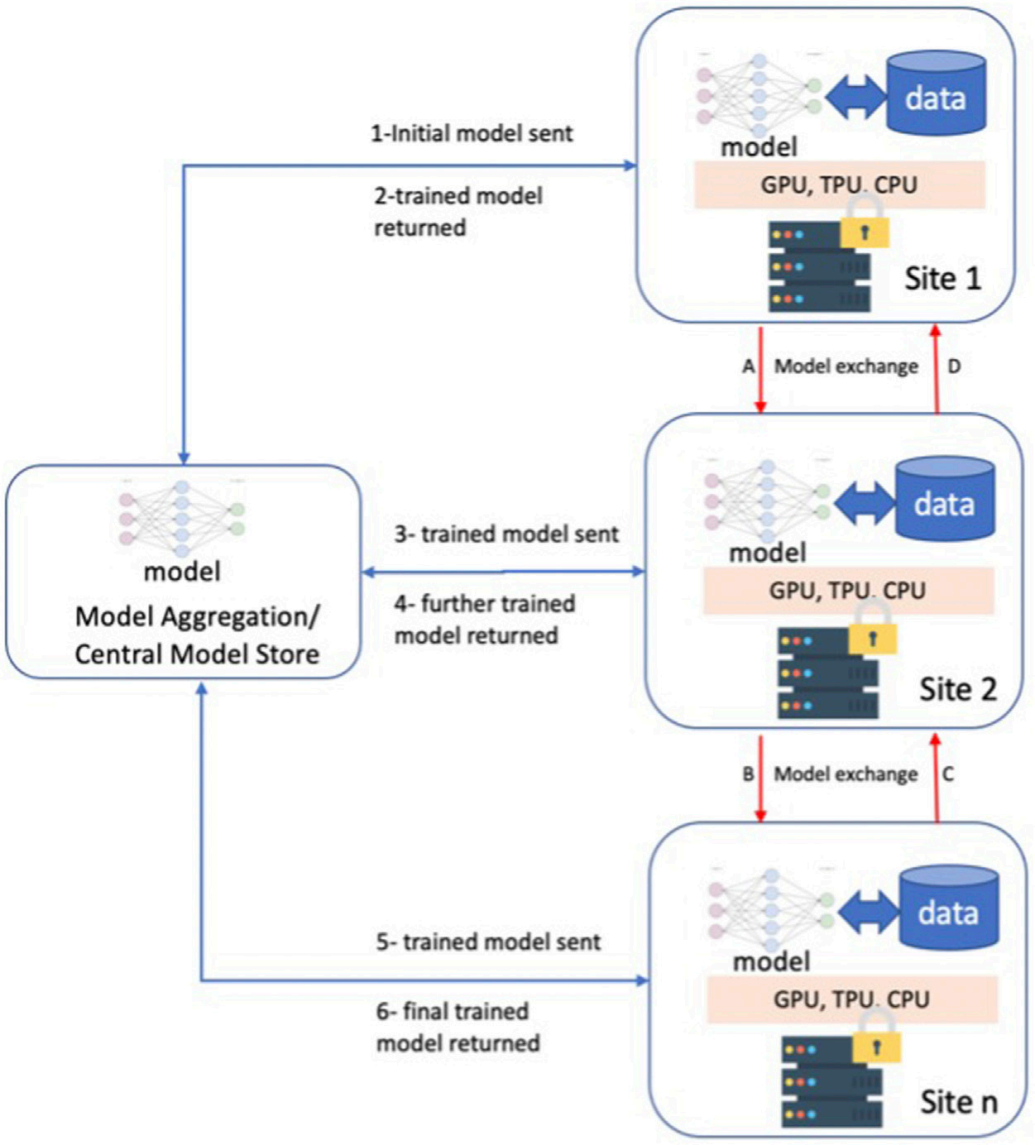
Federated Learning Overview and two different approaches; 1-6 model exchange via a centralized model store, A-D direct model exchange among the participating sites.

Machine Learning (ML) has shown promise to revolutionize the healthcare industry (Topol, 2019; Griffin et al., 2020). From image classification and understanding to natural language processing, ML approaches have had many advancements and, in some cases, surpassed human performance (Esteva et al., 2017; Haenssle et al., 2018). However, data availability and underrepresentation of minorities in healthcare datasets are well-known impediments to ML research (Obermeyer et al., 2019) and lead to relatively low performance for disproportionately represented ethnic groups (Gao and Cui, 2020). Furthermore, distribution discrepancies in training data from these populations result in biases that are one of the major hindrances before generalizing ML approaches. Given the large volume and diverse data needed for model training, Federated Learning (FL) approaches may provide a novel opportunity for the future of ML applications (Rajendran et al., 2021; Sarma et al., 2021). FL is a collaborative ML training approach in which training data is not centralized and stays within organizational boundaries (Figure 1).

FL has been in use for years in other domains (e.g., cellular phones, etc.) and recently has drawn attention in healthcare. One of the early implementations of FL in biomedicine is the “privacy-preserving distributed algorithm to perform logistic regression (ODAL) across multiple clinical sites” that has achieved low bias and high statistical efficiency (Duan et al., 2020). The models tested the data on association between medication and fetal loss with the random sampling-based simulation considering privacy and model bias reduction.

The Friedman Curve indicates technological advancement has overtaken present human governing capacity, the only way to bridge the gap is *via* the introduction of rapid problem identification and prudent regulation of FL (Friedman, 2016). Axiomatically, there are several structural issues that need to be addressed for wider utilization of FL.

Protected Health Information (PHI) defines the 18 identifiers that can be tied to an individual and is regulated under the Health Insurance Portability and Accountability Act (HIPAA). Nicholas Carlini et al. (2019) provide evidence that it is possible to reveal sensitive personally identifiable information (PII) as a result of unintended “memorization” by neural networks. Due to the nature of the data that could be memorized by the FL model, it would be appropriate to classify the model under the definition of PHI since there is a risk of the model itself containing the PHI of patients. Doing so would be essential for compliance with the HIPAA Security Rule, which provides specific guidelines for the utilization, employment, and protection of the data containing PHI (i.e., the models in FL). In essence, treating the partially or fully trained model as PHI could limit malicious actors from accessing the potentially memorized portions of neural networks.

Among the issues, training data variations due to data types and their capture quality make data preparation salient to the success of the endeavor. Some of the most relevant clinical information may not be accessible or be recorded incorrectly in a way that is not representative of the studied population or missing. Moreover, data quality challenges in healthcare are an acknowledged barrier to research in general and ML in particular. Having to decide between lacking the ability to investigate the data or possible privacy issues is a difficult dilemma. Despite the perceived and studied benefits, there are some challenges for wider implementation and acceptance of FL that can be categorized under a tripartite division: privacy, ethical, and legal considerations.

## Security and Privacy of the Model Exchange Challenges

Federated Learning was originally proposed as a method of training machine learning models while preserving the privacy of individual contributors. By design, the FL participants send model updates rather than the entire dataset, but these updates may reveal sensitive information. Therefore, the security and privacy of the FL approaches have been studied extensively (Hitaj et al., 2017; Beaulieu-Jones et al., 2018; Li et al., 2019). The primary goal is to prevent malicious intended nodes of a FL implementation from attacking the model to regenerate the training data of a contributor to the model. Additionally, model updates from various institutions must not carry patterns that reveal patients’ PHI.

The transmission of the bare-minimum information required by the model should be set at the discretion of the FL developers in order to limit the potential of PHI being intercepted by a malicious actor (Gad, 2020). Exercising caution is a proactive measure that is not computationally intensive. It would be prudent to synchronize updated information, and disseminate informal standards to proliferate throughout the FL community. Correspondingly, harmonizing the updates so that they are sent simultaneously will limit identification of an update with the respective institution’s information (Rieke et al., 2020).

Fundamental privacy control in FL requires that data never leave the local environment, and that the global model server should receive updates of a local model. However, these local updates are vulnerable to privacy attacks without adequate protection. The main methods of protecting this information in the FL process are global differential privacy, model encryption, and secure multi-party computation (SMC).

*Global differential privacy (GDP)* is defined as a framework in which an algorithm is considered differentially private only when adding a singular instance into the training set does not cause a statistically significant change to the algorithm’s output. With GDP, one cannot conclude if any given singular data sample was used in the model training process (Truex et al., 2019). GDP is the most prevalent method of privacy protection due to its simple algorithmic convenience, information guarantee, and relatively small overhead cost (Li et al., 2020). GDP is accomplished by randomly perturbing the parameters of the local model before aggregation and incorporation into the global model. Including noise is the most frequent type of perturbation, of which Gaussian Noise, Laplacian Noise, and Binomial Noise are among the most popular candidates.

Another useful method of protecting participant information in FL is *model encryption*. In this method, the parameters of the global model are encrypted before being distributed to participating institutions for local training. After local models receive encrypted parameters, they return encrypted local gradients. In some cases, local gradients are further perturbed. The global model then aggregates all local gradients and decrypts them to update the universal model (Xue et al., 2021).

Lastly, *secure multi-party computation* is a type of privacy preservation method which requires that only a trusted number of parties are permitted to obtain the output of a function using the input of their own private data and are prevented from knowing anything about the model other than this output (Truex et al., 2019). One way to achieve such privacy is with *homomorphic encryption*, where local model updates are masked and not decrypted for aggregation. Trusted parties perform computations while the model is encrypted, revealing no information about the global model (Phong et al., 2018; Sav et al., 2020; Zhang et al., 2020). Privacy-preserving platforms have been proposed that use a hashing framework to represent patients across institutions with hash codes rather than revealing information. This model applies homomorphic encryption to patient similarity searches (Lee et al., 2018). Despite its potential benefit, the additional computational burden and having insufficient supporting frameworks and libraries make homomorphic encryption not easily implementable (Kim et al., 2018).

### Vulnerabilities of the Proposed Solutions

**FIGURE 2:**
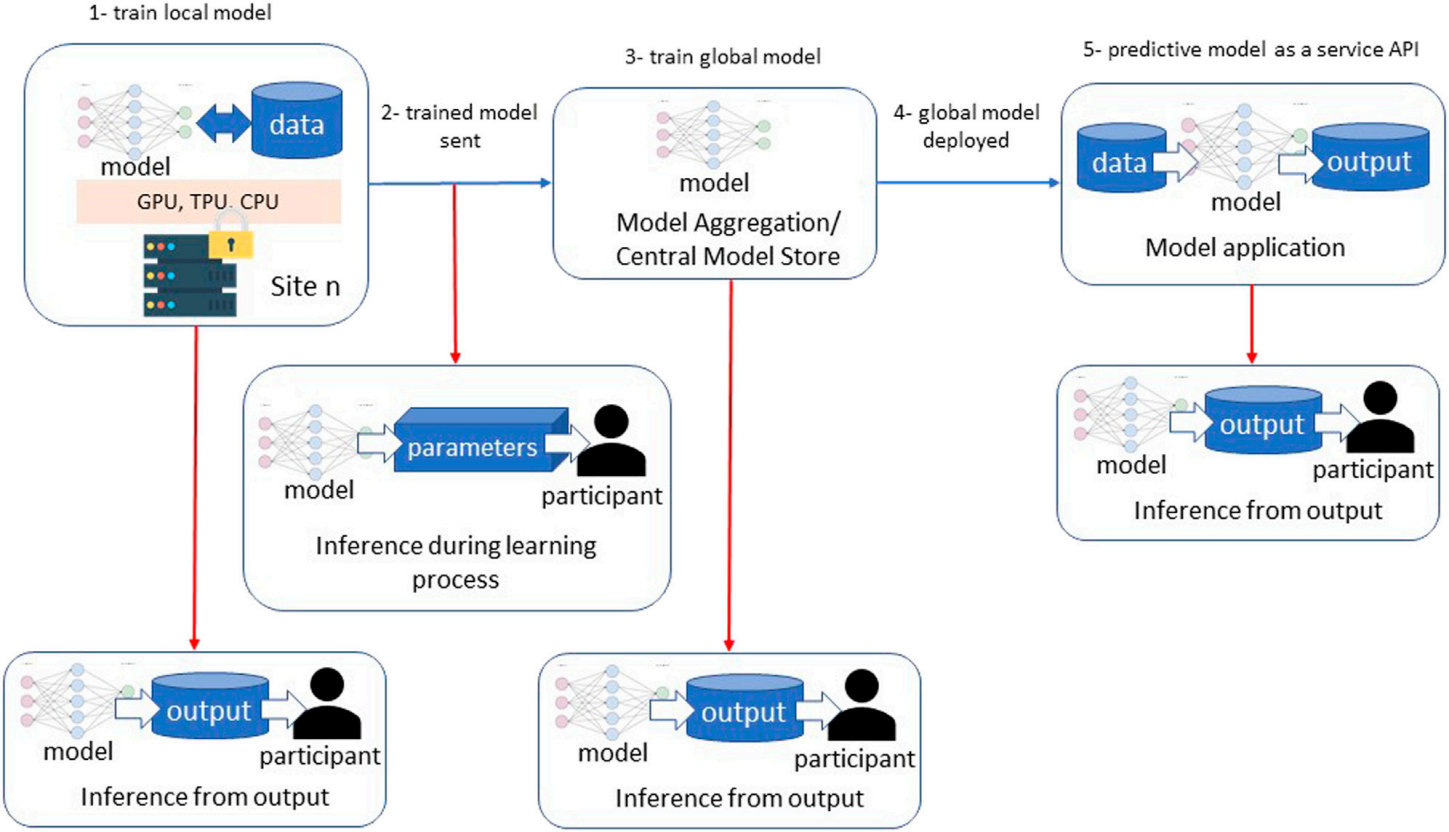
Privacy vulnerabilities in federated learning processes.

Although GDP and model encryption have made significant progress in keeping patient data safe, utilizing either method will not make FL models immune to privacy breaches. Figure 2 depicts the two main types of data leaks and privacy breaches in federated learning, which are: *inference during the learning process* and *inference over the output* (Truex et al., 2019). *Inference during the learning process* is accomplished when a member of the federation infers information about a participant of the local model when the local model’s parameters are transferred to the global model. In other words, this attack requires white-box access. Hitaj et al. (2017) have used a Generative Adversarial Network (GAN) to generate samples of participant data that are indifferentiable to a discriminative model from the original participant data used in model training including reconstructing images. Such an attack might be used to de-identify patient data in a medical application of FL (Hitaj et al., 2017).

*Inference over the output* takes place when a user has black-box access to the predictive model. In this attack, a user compares input and model output in order to infer if a particular sample was used in the model training. Shokri et al. (2017) demonstrated a Membership Inference Attack against a FL model with white-box access by building an attack model that recognizes the difference between output from data the model was trained on versus data the model encountered for the first time. Such an attack could expose information about patients used to train a local or global FL model.

The underlying trend among FL models that suffer inference over output privacy attacks is that the more overfitted a model is, the more vulnerable it is. Consequently, the more diverse the training set is, the better it generalizes and subsequently is less likely an inference over output will succeed (Shokri et al., 2017).

Research in preventative measures for security attacks focuses on combining GDP, model encryption, and additional privacy control features. GDP is used to combat inference over output attacks, while model encryption prevents inference during the learning process attacks by keeping model parameters confidential. Enhancements to these main methods include:

1. Local differential privacy (LDP), a type of differential privacy, where each data-supplying client perturbs information locally and sends a randomized version to the global server, protecting both the client and server side from information leakage (Wei et al., 2020)
2. Gradient-based learning methods, in which gradient responses are clipped to limit the influence of each on the overall update (Li et al., 2020)
3. Selective parameter sharing, in which a fraction of an asynchronous local update is communicated to the global server (Shokri and Shmatikov, 2015)
4. Secure aggregation where only the weighted averages of the update vectors from a random collection of one or more contributors are used to update the global model–another realization of SMC (Bonawitz et al., 2017; Bell et al., 2020).
5. Sparse vector technique, where a clipped and noisy component of an update must fulfill a noisy threshold in order to be included in the local update (Li et al., 2019)
6. Cyclical weight transfer, in which models are trained at one institution at a time for a predetermined number of epochs before being transferred to the subsequent institution (eventually cycling back to the first institution) in order to quantify and minimize the amount of information an individual contributes (Beaulieu-Jones et al., 2018)
7. K-random sampling of clients to participate in each model aggregation (Wei et al., 2020)

It is important to note that there exists an inherent trade-off between data perturbation and model accuracy. Adding more noise with GDP results in greater confidentiality, but may significantly compromise the model’s predictive accuracy (Truex et al., 2019). Researchers have used combinations of the methods above in order to reduce the amount of noise necessary to satisfy the definition of GDP. For instance, Truex et al. (2019) combined SMC with GDP to reduce noise and improve model efficiency and accuracy by requiring each trusted party to add noise to encrypted responses before communicating them to the aggregator.

Securing complete privacy in FL remains questionable, as researchers have been able to develop successful attack models that leak PII (Hitaj et al., 2017; Shokri et al., 2017). In the case of healthcare data, it is essential to eliminate the potential of privacy breaches as such data is especially sensitive.

## Ethical Perspectives

Ethical behavior has long been an integral aspect of the practice of medicine, with Hippocrates enshrining the notion of medical ethics in his seminal works (Bujalkova, 2001). The numerous technical challenges of FL discussed oftentimes have multiple solutions, and part of the decision-making calculus necessitates the inclusion of ethics in order to respect the humanity of the patients that healthcare workers seek to heal. Consistent conversation about the ethical foundation will enable a culture of respect that prioritizes the patient and their human rights (Markose et al., 2016).

Professional societies such as the American College of Radiology (ACR), along with several other U.S. and international radiology organizations, have released an exemplary consensus and guidance document on the importance of developing ethical standards for ML (Geis et al., 2019). Toolkits such as the ACR’s AI-LAB that promote a vendor-neutral framework to develop algorithms based on patient populations may also allow extensibility of algorithms (Allen et al., 2019). Even supranational organizations such as the United Nations have begun discussing the intersection of AI, ethics, and health, striving to establish a “Global Dialogue” for these newly recognized concerns (Azoulay, 2019). The WHO has recently released a guideline from various industry experts, academics, and public sector officials with an emphasis on the protection of human autonomy, equity, transparency, and sustainability which is indicative of a greater trend by the UN to encourage mindfulness in regard to ML (WHO, 2021).

Potential biases that may arise due to disproportionate representation of minority groups is one of the endemic problems that arise from current ML, commonly referred to as “Algorithmic Discrimination” (Köchling and Wehner, 2020). For example, Google’s facial recognition algorithm was widely criticized for its appalling identification of Black people as apes in 2015; they promptly “fixed” the issue by preventing the algorithm from classifying gorillas (Mulshine, 2015). Even 6 years later, ML still presents problems in healthcare, with melanoma detection algorithms being primarily trained on light-skinned individuals; Black people, while less likely to develop melanoma, are more likely to die from it (Noor, 2020).

## Legal Considerations

Due to variations in legal definitions and corresponding regulatory frameworks, the clinically focused applications [e.g., Clinical Decision Support (Guidance, 2019), diagnostic tools] have additional requirements set forth by the Food and Drug Administration (FDA). The United States presently lacks the requisite federal legislation to prudently govern the use of PII in relation to Artificial Intelligence, and consequently, FL. While there is an initiative on the part of private corporations towards establishing guidelines, ML and FL are not thoroughly regulated. This is partially due to the relative novelty of modern ML as well as the difficulty of establishing efficacious legislation on a rapidly developing technology.

The two prior administrations in the United States, (the Obama and Trump Administrations) saw, for the first time in 2016, the executive branch provide insight into the burgeoning field of administering artificial intelligence (Technology NSaTCCo, 2016; Policy TWHOoSaT, 2018). While the two administrations inevitably differ on their objectives in regard to policy, these directives are indicative of a greater trend towards legislating AI with greater frequency. Congress has also passed legislation involving AI, however, the lion’s share was directed towards autonomous vehicles or the Department of Defense and not AI in the healthcare sector (Caribbean RoAITAat, 2021).

HIPAA, which was initially enacted in 1996, and the Health Information Technology for Economic and Clinical Health Act (HITECT) of 2009 have permeated every aspect of healthcare. While comprehensive for the time, AI and FL as a subset pose sui generis concerns, which necessitate an update to factor in the proliferation of ML in healthcare (Cohen Healthcare, 2021). A myriad of entities such as the Center for Open Data Enterprise and the Department of Health and Human Services have published roundtable reports, which proposed numerous recommendations of action in regards to the intersection of PHI and AI (Enterprise TCfOD, 2019). Leveraging expertise and the experience gained from the incorporation of ML into healthcare as well as inspiration from the European Union’s (EU) laws will be critical in updating HIPAA after 25 years.

Other issues that are inherent in ML currently are: lack of transparency for algorithms, lack of contestability for non-optimal results, the legal status of AI with questions of “personhood,” and correspondingly, a lack of accountability for damage that may arise from the algorithm (Rodrigues, 2020). Matters of liability for damages form an ancient pillar of the law and are continuously contested. AI is no exception. In the case of ML algorithms, the damage is not directly attributable to the algorithm itself as it is not considered a “person” and thus provides complications for a claimant seeking to extract compensation from a liable party. In the case of FL, if, as a result of the model revealing some form of PHI through reverse engineering, it would be considerably more difficult to establish the culpable party amongst multiple institutions. A potential solution, as Hallevy would suggest, would be to hold AI accountable in a similar vein to corporations and propose different punishments for these entities (Hallevy, 2015).

### *In Vitro* Diagnostic and Clinical Decision Support

It is critical to have rigorous change protocols and data provenance for algorithm modifications to ensure safety and provide transparency to users during updates to algorithms in real-world clinical settings. However, these may not be routinely implemented. While ML-based CDS tools can assist in automated detection, classification, or reporting, safeguards are essential to support decision-making and to proactively mitigate potential errors that may arise from these complex systems. Model updates should be kept minimal for the ML models for CDS or similar clinical use. For instance, the “black-box” nature of many ML algorithms makes interpretation and benchmarking performance difficult. To improve algorithm transparency, Price et al. proposed a three-step framework for validating “black-box” algorithms, which involves: 1) having high quality training data and development procedures, 2) testing algorithm performance against independent test data, and 3) evaluating performance continuously (Price, 2018).

The FDA has released several policies to ensure safe and effective use of ML-based software for medical purposes, including regulatory frameworks for software as a medical device (SaMD, 2019), clinical decision support (Guidance, 2019), and a pre-certification program (FDA, 2021).

## Contribution Equity

When two institutions are training a single ML model, there exists a tradeoff between the privacy of the data and the joint effort of model development (Rieke et al., 2020). Because the training data will be decentralized it would be beneficial to have them coordinate on tasks such as harmonizing data. A potential solution towards the matter of privacy that arises from having a decentralized set of data is to limit the number of coordinators or to decline consuming data not explicitly related to the model.

Machine learning algorithms may be profitable once validated and implemented such as CDS. Within a FL framework, the unique nature of having the data separated entails concern about an equitable distribution of profit. Shapley values are a well-established method for determination of equitable payoff, yet in FL they pose a computationally prohibitive and horizontally limited applicability due in part to its lack of ability to be extrapolated (Huang et al., 2020; Wang et al., 2020). Yet, the nature of FL does warrant an evaluation of the utility it poses as well as any derivative models that can be created such as FedCoin (Liu et al., 2020). Essentially, in the case of two entities, if one of them supplies data that is less desirable (such as incomplete or plentiful data) and the other entity provides more heterogenous and usable data, deciding the value of each contribution is vital. Due to the critical nature of data within the healthcare sector, vigilance regarding efficacious incentivization of data is invaluable. Predetermined measurements should be agreed upon in a contractual arrangement by the parties that will take part in a FL project to mitigate any disputes or issues that may arise from this situation. To avoid difficulty that may occur, it could prove beneficial for a standard to be agreed upon for proportionally dividing up the profit depending on prospective criteria, namely the completeness, heterogeneity, quantity, conformance, and provenance of data. (Rieke et al., 2020).

### Conceptual Considerations for Assessing Data Contribution

**FIGURE 3:**
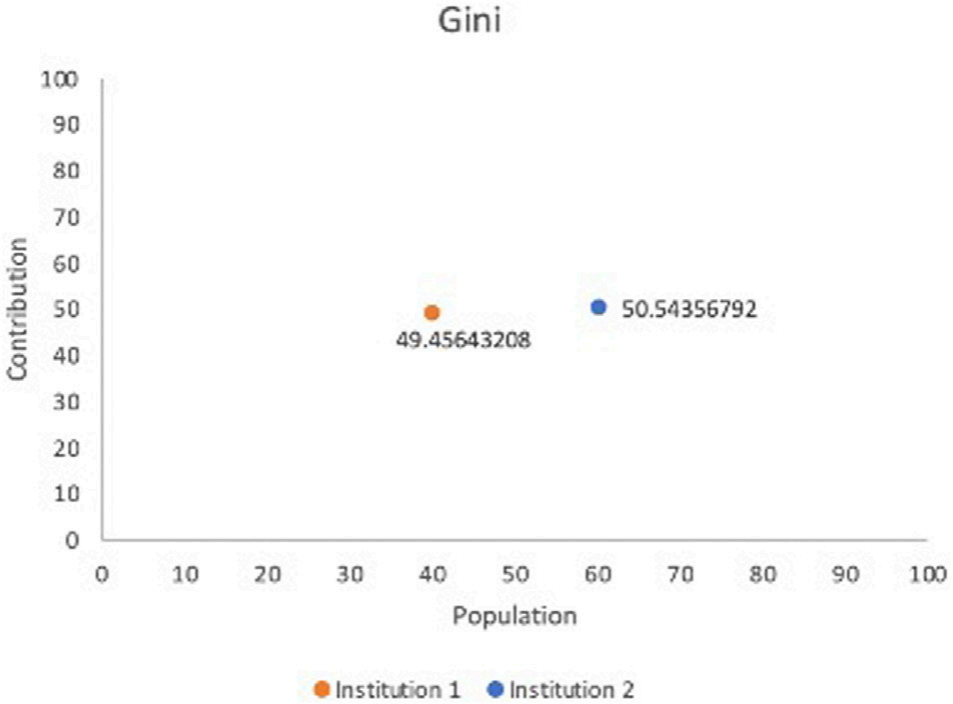
Gini representation based on model contribution and data population.

We propose a conceptual model and the below equation that, we believe, can be employed to gauge levels of involvement in a FL framework. Due to the unique and distributed nature of FL, it would be advantageous to have a standard evaluation strategy for purposes such as determining remuneration for the decisively engaging factor of data. This model was partially inspired by the Gini Coefficient [Gini Index (2008). The Co, 2008], which determines income inequality; with influence from the Sørensen–Dice Coefficient as a way to determine the relative value of the data from its’ similarity (Figure 3). The least similarity would be viewed favorably and incentivized accordingly. Even though the Information Gain Function could be considered as an alternative, Raileanu et al. have proved that the Gini Coefficient only disagrees by 2% with Information Gain in all cases (Raileanu and Stoffel, 2004). Through establishment of a model in which the data is evaluated for its’ heterogeneity, the greatest differences will be incentivized and prioritized as opposed to data with a more uniform quality. In a similar vein, we advocate the determination of the level of participation *via* data contribution. Prioritizing timeliness while emphasizing the importance of data is a philosophy which enables institutions to be ethical stewards while not sacrificing speed to have the participants focus on the main effort of research as opposed to extensive contractual negotiation and timely calculations.

*Mi* = Model development contribution level of *ith* site: Mi *ɛ* ℝ = [0,1]
*Di* = Data contribution level of *ith* site: Di *ɛ* ℝ = [0,1]
*N*= Number of data records in a FL framework

$$\mathrm{Model}\;\mathrm{Contribution}\;\mathrm{of}\;ith\;\mathrm{node}=\frac{M_{i}}{\sum_{i=0}^{n}M_{i}}.$$ (1)

$$\mathrm{Data}\;\mathrm{Contribution}\;\mathrm{of}\;ith\;\mathrm{node}=\frac{D_{i}}{N}.$$ (2)

Our test case employed the Breast Cancer Wisconsin Dataset^1^, which consists of 569 records and the 30 features include radius, worst concave points etc. We randomly split the dataset into two institutions to simulate a FL cooperation; institution 1 possesses 227 records and institution 2 has 342 to mimic a horizontal FL operation. Initially, we have calculated the Sørensen–Dice Coefficient: institution 1: 0.712854305 and institution 2: 0.728524045 and computation took 0.007 and 0.004 s respectively. Table 1 shows normalized Sørensen–Dice Coefficients and percentage of record/patient population by each institution. The Sørensen–Dice model provides a far simpler and computationally inexpensive approach for evaluation of both datasets with regards to a horizontal FL model.

**TABLE 1:** Sørensen–Dice Coefficient calculated model contribution percentage and percent population contribution.

	Population	Model contribution
Institution 1	0.49456432	0.39894552
Institution 2	0.50543568	0.60105448

**FIGURE 4:**
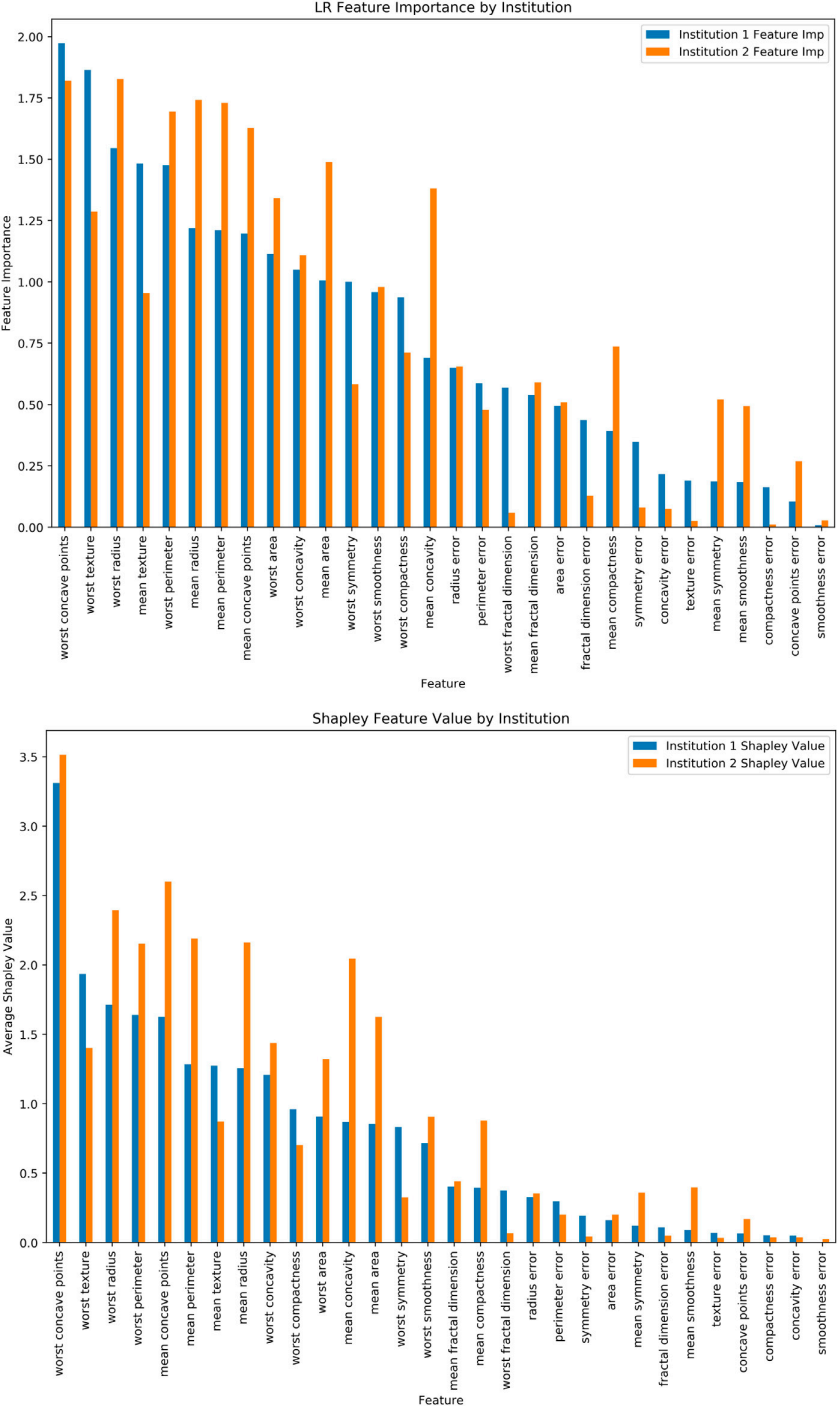
**(A)** Logistic Regression Feature Importance, **(B)** Shapley Values for the same dataset and ML model.

Additionally, we have run Logistic Regression (LR) across the “institutions” and feature importance for the LR Coefficients (Figure 4A) and Shapley Values for the same LR model (Figure 4B). It should be noted that features have varying levels and orders of importance for both approaches. Furthermore, it took 1.401 s per record (i.e., 797.169 s) for the computation in this relatively smaller dataset. The Shapley values, while traditionally seen as an appealing method due in part to its familiarity, faces limitations regarding the computational burden that it places as well as the limited applicability to horizontal FL operations which have been widely used within the healthcare focused FL implementations (Molnar, 2019).

### Data Contribution Considerations (*Di*)

The initial and possibly one of the most important steps after identifying a realistic problem, is data collection. Although nowadays healthcare systems collect more data than ever, data capture and the way data are stored varies considerably, presenting a non-trivial challenge. Virtuous management and collection of data is a prerequisite for the ethical responsibilities an institution possess towards its’ patients with the WHO advocating for accountability and mindfulness for integration of ML in healthcare (WHO, 2021). Similarly, without conscious adherence towards legal obligations, a model trained may yield an algorithm which is discriminatory and thus a violation of different laws such as the Equal Protection Clause, however, more precise legislation is needed to be more comprehensive and effective against instances of discrimination (Li, 2021). The Shannon’s Information Theory (Entropy) (Shannon, 1948) could be one of the quantification models that may work for data contribution assessment purposes. The components are:

Data Completeness: The completeness of data provides an analysis of the data and for any absences within it, irrespective of the values that are present. Functionally, Data Completeness is an important measure for the conformity of the model (Kahn et al., 2016).

Data Heterogeneity: We sought to include Data Heterogeneity as one of the principal components for determining Data Contribution due to the significant legal and ethical issues of Algorithmic Discrimination present in ML. In essence, institutions with greater diversity in the data that they contribute will be rewarded, with the hope that this will encourage further inclusion of minority and marginalized populations. The Sørensen–Dice Coefficient (Sørensen, 1948) could be used here to determine the similitude of the data as demonstrated by Table 1 and Figure 3.

Data Quantity: The amount of data to be used in the model training and validation (e.g., number of records or number of patients etc.).

Data Conformance to the Model: Conformance in conjunction with Completeness will ensure that the data will comply with the standards required by the model to ensure the usability of the data. The three subcategories are value conformance, relational conformance, and computational conformance all of which are invaluable in reliable and successful utilization of the model.

Data Provenance: Provenance in this framework refers to the lineage of data. The data possessing proper documentation is conducive to many institutions’ commitment to transparency in the healthcare field. Data provenance is particularly important for those ML models that will seek FDA approval/certification. The FDA would like to ensure that the source and provenance information around each ML result are properly captured, recorded, and carried along with the associated data.

### Other Factors

There are numerous other considerations that we did not include in the model due to the extreme variability of the following factors: Data Extraction and Preparation Cost. Additionally, model development cost which refers to the labor and efforts behind the creation of the algorithm is an important yet highly fluid factor which demands extensive exploration in its own right. Notwithstanding, the aforementioned are all dependent upon the specific circumstances of the institution and will contribute to volatility in determining equity. For example, Data Extraction Costs for one institution may be extensive whereas another institution may be able to have data for free. We do not want to penalize institutions for similarly volatile scenarios and will thus omit them.

## Conclusion

Establishing an agreed upon definition of data privacy between each of the collaborating parties is critical in order to standardize potentially incongruous definitions or privacy expectations. Reducing the risk of conflict with this synchronization will benefit the FL project as a whole. An alternative could also be to have each of the *n* institutions officially accept and agree upon their respective institutions’ definitions instead of working to establish a wholly new one for the purposes of this temporary FL project.

Additionally, it would prove beneficial to share the details of the collaboration with the Data Privacy Officer as well as informing them of their respective institutions’ responsibilities within the FL framework so that they may be better able to judge the specific circumstances of the project. The Chief Privacy Officer should be consulted even in institutions when the only contribution will be computational resources or the model. Furthermore, establishing a dialogue between the Institutional Review Board (IRB) of respective entities as well as researchers planning to conduct a FL project is critical to the efficacy of the project and the fulfilment of the hospital’s responsibility for their data. FL is a novel application of ML with significant advantages, while posing justifiable concerns for IRBs. Unfortunately, there is oftentimes a disconnect between the researchers and the IRBs that can be remedied through communication and mutual understanding. In the case of a FL proposal, it would behoove the researchers to demonstrate that their study will align with the aims of the IRB in protecting the PHI of their patients (Lee et al., 2016).

We have chosen to emphasize the data aspect of the FL process as it presents a crucial component of research due to it involving the PII or PHI of patients and being a focal point for legal and ethical risks. Novel and synergetic implementations of FL and contribution equity evaluations -such as the popularization of blockchain technology-in the future may see the paradigm shift towards enabling further adherence to Shapley, yet the simplicity and relative speed of the Gini-influenced Sørensen–Dice Coefficient can be beneficial for potential preliminary proposals or in situations where the rapidity of the system is an acceptable tradeoff for a less detailed system. Shapley values as they stand are not feasible to implement due to being far too computationally expensive.

The proposed model enables participants in a FL collaboration to transparently ascertain contribution in terms of providing data and corresponding feature importance. Quantifying the cooperation between distinct entities provides a noteworthy approach to ease friction that may otherwise occur regarding proper division of financial gain and accountability. However, the model does not account for highly variable computing and data acquisition costs, as the insertion of these considerations would merit further study. Notwithstanding, measuring contribution will prove invaluable and greatly aid the growth of this technology as it reduces a contentious barrier to entry.

## Footnotes

1
https://scikit-learn.org/stable/datasets/toy_dataset.html
